# Emulsion-Based Delivery Systems to Enhance the Functionality of Bioactive Compounds: Towards the Use of Ingredients from Natural, Sustainable Sources

**DOI:** 10.3390/foods12071502

**Published:** 2023-04-03

**Authors:** Júlia Teixé-Roig, Gemma Oms-Oliu, Isabel Odriozola-Serrano, Olga Martín-Belloso

**Affiliations:** Department of Food Technology, University of Lleida—Agrotecnio Center, 25198 Lleida, Spain

**Keywords:** emulsions, delivery systems, bioactive compounds, sustainability, plant-based

## Abstract

In recent years, the trend in the population towards consuming more natural and sustainable foods has increased significantly. This claim has led to the search for new sources of bioactive compounds and extraction methods that have less impact on the environment. Moreover, the formulation of systems to protect these compounds is also focusing on the use of ingredients of natural origin. This article reviews novel, natural alternative sources of bioactive compounds with a positive impact on sustainability. In addition, it also contains information on the most recent studies based on the use of natural (especially from plants) emulsifiers in the design of emulsion-based delivery systems to protect bioactive compounds. The properties of these natural-based emulsion-delivery systems, as well as their functionality, including in vitro and in vivo studies, are also discussed. This review provides relevant information on the latest advances in the development of emulsion delivery systems based on ingredients from sustainable natural sources.

## 1. Introduction

Bioactive compounds such as carotenoids, polyphenols, or polyunsaturated fatty acids (PUFAs) have been found to reduce the risk of cardiovascular diseases, cancer, and other diseases [1,2]. This positive effect has been correlated to the different biological activities of these compounds, which are present in plant-based foods such as fruits, vegetables, tea, and wine, among others [3,4]. Moreover, promising novel sustainable sources of these compounds have emerged in recent years, with microalgae and agrifood residues being sources of high interest. However, most bioactive compounds are prone to degradation and present low bioavailability [5,6]. To overcome these problems, encapsulation techniques can be very useful, as they increase the stability and functionality of these valuable compounds [7,8]. Among them, emulsion-based delivery systems have been widely used, with some examples being nanoemulsions [9,10,11], highly-concentrated emulsions [12], or double emulsions [13,14,15]. These encapsulation systems have been shown to provide high stability to encapsulated compounds, as well as to increase their bioaccessibility and bioavailability. 

Initially, emulsion-delivery systems were obtained mostly by using synthetic ingredients, leading to high stability of the resultant systems. However, in recent years, consumers have become aware of the impact of synthetic ingredients on health and on the environment, increasing the demand for food products containing ingredients from natural sources. For that reason, the design of emulsion-based delivery systems must go forward with the use of components of natural origin, with emulsifiers being included in the main focus. Indeed, most synthetic emulsifiers used in the formulation of these systems have been proven to be associated with health problems and toxic symptoms with a long administration. It has been observed that these emulsifiers could bind to proteins, enzymes, and phospholipid membranes in the human body, producing alterations such as enzyme dysfunction or protein structure modification and phospholipids in the membrane cell [16,17]. In addition, the use of natural ingredients, which are obtained from plants and algae, can help to take an important step towards more sustainable and clean-label products. While synthetic ingredients are associated with environmental issues due to their low biodegradability [18], natural ingredients such as proteins, polysaccharides, phospholipids, or saponins that are obtained from renewable sources can be a feasible alternative [19]. 

Therefore, the aim of this article is, first, to provide a general overview of the most innovative sources from which to obtain bioactive compounds in a more sustainable way. Second, this paper also reviews recent advances in the use of natural emulsifiers to obtain stable emulsion-based delivery systems with optimal functionality.

## 2. Bioactive Compounds 

**Figure 1:**
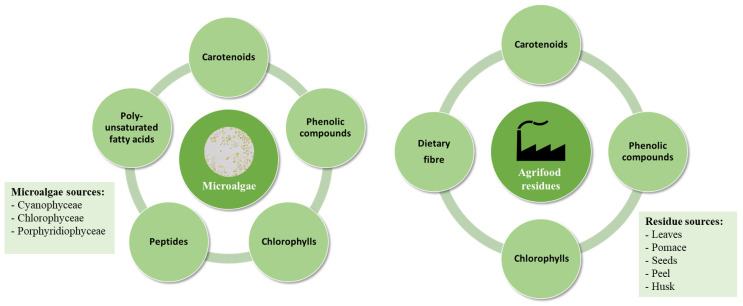
Bioactive compounds from microalgae and agrifood residues.

In recent years, purified extracts obtained from plants, fruits, and vegetables have been used as bioactive compounds, both lipophilic and hydrophilic. However, in recent years, the search for more sustainable foods has focused the attention on the use of alternative sources to obtain bioactive compounds. Among these natural alternatives, the use of microalgae seems to be a suitable source from which to obtain these bioactive compounds due to the fast growth, high yield, and short cultivation time of microalgae [20] (Figure 1). In addition, residues from the agrifood industry are promising sources of these compounds with biological activity. This form of residue valorisation would represent a new conceptualisation of sustainability in the food chain, moving from a linear to a circular economy and allowing the possibility of advancing the use of co-products to generate new and safe value-added products [21].

### 2.1. Novel Alternative Sources of Bioactive Compounds 

#### 2.1.1. Microalgae

These organisms produce metabolites which have been associated with relevant health benefits [22]. In general, these metabolites are produced as a response to environmental stress and include carotenoids, polyunsaturated fatty acids, phenolics, chlorophylls, and peptides, among others.

- Carotenoids: It has been found that microalgae can produce and accumulate carotenoids, with *Chlorophyceae* being the dominant carotenoid-producing group of xanthophylls and carotenes [23]. Specifically, *Dunaliella salina* and *Haematococcus pluvialis* are commonly used for high-value carotenoid production due to their high content of carotenoids such as β-carotene and astaxanthin, which can represent up to 14% of the microalgae dry biomass [24]. Other compounds such as lutein have been identified in *Muriellopsis* sp., although the concentrations were lower (0.4% to 0.6% per dry biomass) [25].
- Polyunsaturated fatty acids: In microalgae, fatty acids cover the largest percentage of total lipids, with polyunsaturated fatty acids (PUFAs) representing 20–60% of the total lipids [26]. *Spirulina* and *Chlorella* are valuable sources of PUFAs such as docosahexaenoic acid (DHA), arachidonic acid (ARA), alpha-lipoic acid (ALA), and eicosapentaenoic acid (EPA) [27]. Microalgae can represent an interesting vegan source of fatty acids that, up to date, have been obtained mostly from animal sources such as fish oil.
- Phenolic compounds: Although high concentrations have been observed in macroalgae, microalgae such as *Chlorella* or *Arthrospira* have been found to contain appreciable levels of phenolic compounds. However, according to the literature, the concentration in microalgae present significant variations due to species type, cultivation conditions, and techniques used for extraction, identification, and quantification [22].
- Chlorophylls: These natural green pigments are crucial in photosynthetic organisms for harvesting energy from sunlight and can be classified as a, b, or c [22]. However, chlorophyll c is present only in brown algae and not in green algae. Among microalgae species, *Chlorella* is the main producer of chlorophyll, with other species such as *Spirulina* and *Arthrospira* producing limited concentrations [28].
- Peptides: Microalgal proteins have been demonstrated to be a source of bioactive peptides after enzymatic hydrolysis. Due to their differentiated sequential, structural, and compositional properties, microalgae peptides exert a list of positive health effects such as antioxidant, antihypertensive, antitumor, and immunomodulatory effects [27,29].

In summary, microalgae are a promising source of multiple bioactive compounds that can be isolated via various extraction methods. Moreover, they can grow in non-potable water and agriculturally non-productive land, in addition to presenting greater surface productivity and photosynthetic efficiency compared to terrestrial crops [30,31].

#### 2.1.2. Co-Products from the Agrifood Industry

During the various stages of food production, the agrifood industry generates large amounts of residues which contain bioactive compounds that present beneficial biological activities for human health [32,33,34]. The main bioactive compounds that can be obtained as agrifood co-products can be classified into carotenoids, phenolic compounds, chlorophylls, and dietary fibre.

- Carotenoids: Various agrifood residues such as tomato peel [35], guarana peel [36] or peel, and the pulp of citrus fruits [37] have been found to contain carotenoids such as lycopene, β-carotene, or lutein. According to these authors, these agrifood residues can contain variable concentrations that can be up to 60% carotenoids per unit of dry weight.
- Phenolic compounds: A variety of phenolic compounds such as flavonoids, phenolic acids, and lignans has been found in different agrifood residues from fruit and vegetables. The waste parts in which they have been identified include pomace [38], leaves [39], seeds [40], peel, or husk [41].
- Chlorophylls: These green pigment compounds, which can be classified as chlorophyll a, b, or c, have been found in residues from different vegetables, especially in the leaves. As an example, chlorophyll a and b, in concentrations ranging from 1132.33 to 1795.93 ppm, were detected as co-products from olive leaves [42,43]. However, higher concentrations have been detected in the leaf residues from broccoli [44] (up to 4477.9 µg/g dry weight) or asparagus [45] (up to 5096 µg/g dry weight).
- Dietary fibre: Both soluble and insoluble fibre have been found in residues from vegetables such as artichoke, carrot, or pepper [46], as well as fruits such as guava or passion fruit [47]. However, most of these residues contained higher amounts of the insoluble fraction rather than the soluble one. In addition, diverse cereal residues have been found to contain soluble (beta-glucans) and insoluble dietary fibres (such as cellulose or lignin) in variable concentrations [48].

Therefore, several bioactive compounds can be obtained as co-products of agrifood residues of diverse origins such as vegetables, fruits, or cereals. The use of these residues as co-products implies an improvement in the production process, focusing it on a circular economy of high value for more sustainable production.

### 2.2. Extraction Methods of Bioactive Compounds

Many extraction methods have been developed to isolate bioactive compounds from various matrices. Among them, there are some called “conventional extraction methods”, which have been widely used to obtain these valuable compounds. Some of them are Soxhlet, maceration, hydrodistillation, infusion, or decoction. However, these methods present some limitations such as the thermal destruction of compounds, the use of high amounts of solvent, long extraction times, and a negative impact on the environment. To overcome these challenges, novel methods that are also called “green extraction techniques” have been developed. These methods provide better environmental, health, and safety properties since they use lower temperatures during extraction, shorter times, higher extraction yield, and better extraction efficiency [49]. Some of the recent technologies that have shown less solvent and energy usage are ultrasound-assisted extraction, hydrodynamic cavitation-assisted extraction, microwave-assisted extraction, supercritical fluid extraction, liquid biphasic flotation extraction, cloud point extraction, pulsed electric field, high voltage electrical discharge, and instant controlled pressure drops [50]. Moreover, it has been found that the hybridisation of extraction by combining two or more green extraction technologies provides excellent separation of bioactive compounds. 

It is well known that the efficiency of the extraction depends on several factors, including extraction technique, plant component matrix properties, extraction solvent, temperature, pressure, and time, among others [51]. Therefore, the extraction methods differ greatly depending on the matrix where the compounds have to be isolated. In general, conventional methods are the most widely used, although in recent years, the use of green technologies such as ultrasound-assisted extraction [52], microwave extraction [53], or supercritical CO_2_ extraction has increased substantially. By using these techniques, the extraction of bioactive compounds from microalgae and agrifood residues has been shown to be highly efficient, and the extraction times have been reduced. Moreover, there are other technologies such as liquid biphasic flotation, pulsed electric fields, and high-voltage electrical discharge that are being investigated and have shown promising results, although some of them are still under lab-scale research [50].

## 3. Emulsion-Based Delivery Systems to Carry Bioactive Compounds

**Figure 2:**
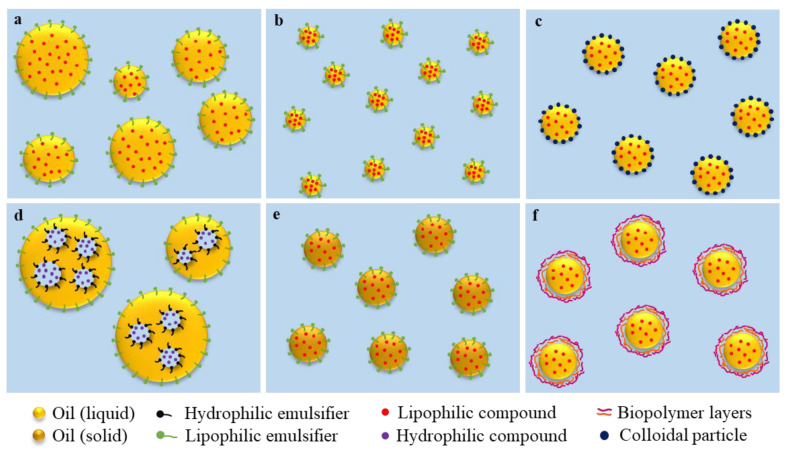
Different emulsion-based delivery systems to carry bioactive compounds. (**a**) Oil-in-water emulsions. (**b**) Oil-in-water nanoemulsions. (**c**) Pickering emulsions. (**d**) Water-in-oil-in water double emulsions. (**e**) Solid-lipid nanoparticles. (**f**) Multi-layer emulsions.

As mentioned, bioactive compounds are easily degraded by multiple factors, so various emulsion-based delivery systems have been investigated to protect them. One of the most relevant properties of these systems is that they need to present high biocompatibility. This means that they cannot be toxic, but they should also fulfil a planned role in the biological environment [54]. These systems can be classified depending on their structure into different groups (Table 1 and Figure 2), with some of the most used systems to encapsulate bioactives as follows:

Simple emulsions and nanoemulsions. Emulsions consist of two immiscible liquids, with one of the liquids dispersed as small spherical droplets in the other, and they have been used to encapsulate different bioactive compounds such as vitamin E [55] or curcumin [56], among others. Nanoemulsions, which are emulsions containing nanometric-size oil droplets ranging from 50 to 500 nm, have been shown to present higher stability over time and higher digestibility than conventional emulsions due to the greater surface area exposed to intestinal enzymes [57,58]. These systems have been widely studied to increase the bioavailability of poorly soluble drugs such as carotenoids [59,60] or vitamin D [61] because authors have found that after reducing the particle size of fat globules, the drug solubility and absorption of the encapsulated compounds was greater [62]. Moreover, nanoemulsions have been shown to have increased encapsulation stability during storage compared to conventional emulsions due to their more stable structure [63]. Simple emulsions can be formed by using low-energy or high-energy methods, with the latter being the most used to produce emulsion-based delivery systems. Low-energy methods (phase inversion temperature method, phase inversion composition method, etc.) are based on the spontaneous emulsion formation under specific system compositions or environmental conditions. In contrast, high-energy methods are based on the use of intense mechanical forces to break up droplets into smaller droplets and are performed by using homogenisers such as high-shear mixers, high-pressure homogenisers, colloid mills, ultrasonic homogenisers, and membrane homogenisers. Nanoemulsions have been used to encapsulate bioactive compounds with low bioavailability to be administered via different routes such as parenteral, oral, nasal, or topical routes. Moreover, they have also been used as edible coatings and to encapsulate flavouring agents or preservatives in food products and have also been applied in the cosmetic and pharmaceutical fields [64].

Double emulsions. These emulsions belong to the group of multiple emulsions since they contain an emulsion structure with coexisting water-in-oil (W/O) and oil-in-water (O/W) morphologies. Double emulsions can be prepared via a one-step or two-step emulsification procedure, with the latter being the most used. In the one-step emulsification method, strong mechanical agitation is required to induce phase inversion. In contrast, in the two-step emulsification method, the first step consists of the formation of a simple emulsion by using high-shear devices including ultrasonicators, high-pressure homogenisers or microfluidizers, among others. Afterwards, the third phase is added, and by using the same devices as in the first step, the double emulsion is formed [65]. Due to their structure, these systems allow the encapsulation of a lipophilic compound and a hydrophilic compound within the same emulsion, which can be an interesting strategy to encapsulate compounds with synergic activity [15]. These systems have been applied to encapsulate labile and/or bioactive compounds with low bioavailability [66,67]. Moreover, using double emulsions has been found to be an interesting strategy to produce fat-reduced products. However, these systems are sensitive and unstable, which can decrease the encapsulation efficiency. The main reasons for their instability are the free energy at the droplet level, osmotic pressure, and Laplace pressure [68]. To improve the stability of these systems, numerous strategies have been developed, such as adjusting the internal and external osmotic pressure by adding sugar or salts or increasing the viscosity [69]. 

Multi-layer emulsions. These emulsions present a simple emulsion structure (O/W or W/O) surrounded by multiple layers of biopolymers. For their preparation, the primary emulsion can be obtained by using the same methods that are used in simple emulsions. Afterwards, biopolymer layers of opposite charges, which act as a stabiliser, are normally constituted by the layer-by-layer electrostatic deposition technique [70]. These systems, which have been reported to contain two, three, or up to four layers of oppositely charged biopolymers, are prone to instability phenomena, with the solution pH being an important factor to prevent them during and after the formation of the interface [71,72]. To prevent instability processes such as bridging flocculation or depletion flocculation, which are observed when there is either an excess or lack of polyelectrolytes in the solution, different strategies can be employed: (1) the saturation method, to empirically determine the biopolymer concentration required to cover the oil droplets; (2) the centrifugation method, to remove the excess of non-adsorbed biopolymers by centrifuging; or (3) the filtration method, whereby the excess of non-adsorbed biopolymers is removed via membrane filtration [73]. The application of multilayer emulsions has been limited compared with other emulsion-based systems. Few studies have investigated its potential to increase the bioavailability of bioactive compounds or control their release. However, due to their structure, they could be very useful in achieving concrete functional performances, offering a high level of control in triggering the release of the encapsulated compounds.

Pickering emulsions. These encapsulation systems can present a simple or multiple emulsion structure and are not stabilised by surfactant molecules but by solid colloidal particles, either organic or inorganic, that should be partly wetted by oil and by water [74]. As an example, they can be stabilised by using turmeric granules [75] or protein-based particles [76]. To prepare Pickering emulsions, all emulsification processes that are used to prepare emulsions stabilised by surfactants can be applied. However, the most commonly used are rotor–stator homogenization, high-pressure homogenization, and sonication. Moreover, other techniques such as membrane emulsification and microfluidic emulsification have been recently applied. These emulsion-based systems present high stability and high biocompatibility without the addition of surfactants. Even under high stress, the shells covering the emulsion droplets have been found to remain in the systems [77,78,79]. Moreover, they present an adjustable permeability, meaning that the release of the encapsulated bioactive compounds can be controlled under the action of external factors such as ultrasonic waves [80]. Among the applications of Pickering emulsions, the encapsulation and protective storage of sensitive or low-bioavailable compounds have been extensively investigated. Other applications include the construction of porous materials, three-dimensional (3D) printing, and their use as a substitute for partially hydrogenated oils [76].

Solid-lipid nanoparticles (SLN) and nanostructured lipid carriers (NLC). SLN are simple emulsions that contain a solid lipid core. These systems are usually formed by using high-energy methods including the hot homogenization technique or cold homogenization technique. For the hot homogenization technique, the lipid is mixed with a hot aqueous solution by using a high-shear mixing device and treated with a high-pressure homogeniser above the melting point of the lipid to obtain nanoparticles. For the cold homogenization technique, the lipid is cooled and crushed into lipid microparticles that are dispersed in a cold aqueous solution. The mixture is then treated with a high-pressure homogeniser at room temperature or below to obtain nanoparticles. SLN have been shown to present some advantages over systems that contain a liquid oil phase, as SLN provide higher chemical stability of the encapsulated compound and are more stable against lipid coalescence. Moreover, these systems may allow a controlled release of compounds since the drug mobility in a solid lipid should be considerably lower compared with a liquid oil [81]. Indeed, the application of these systems as carriers of labile and low-bioavailable compounds has been tested in vitro and in vivo [82,83]. However, SLN are prone to aggregation and present a highly ordered structure of the crystals within that can promote the expulsion of the encapsulated bioactives, making them more prone to degradation [84]. To overcome these problems, NLC containing liquid lipid mixtures rather than pure solid lipid have been developed. In these systems, the mixture of lipids generates a less ordered crystal structure that allows accommodating more lipophilic bioactive compounds, increasing the encapsulation efficiency and reducing the adverse excretion caused by the polymeric transition [85].

**Table 1:** Preparation techniques, advantages, and limitations of different emulsion-based delivery systems.

System Type	Preparation Techniques	Particle Size	Advantages	Limitations
Conventional emulsions	- High-energy (high-pressure homogenization, sonication, microfluidization). - Low-energy methods (self-emulsification, phase inversion, membrane emulsification).	500 nm–100 µm	- Ease of preparation. - Low cost. - These emulsions can be used in their wet state or be dried to form powders, facilitating their transport and utilisation in some applications.	- Prone to physical instability when exposed to environmental stresses such as heating, freezing, drying, pH extremes, etc. - Limited control over the ability to protect and control the release of the encapsulated compounds.
Nanoemulsions	- High-energy (high-pressure homogenization, sonication, microfluidization). - Low-energy methods (self-emulsification, phase inversion, membrane emulsification).	<500 nm	- They can be easily incorporated into food systems since they are usually optically translucent. - Higher physical stability than conventional emulsions to gravitational separation and aggregation. - Increased surface area compared with conventional emulsions that enhances the digestibility, compound bioaccessibility, and bioavailability. - Higher encapsulation efficiency and compound stability during storage time.	- Some of the synthetic emulsifiers used to obtain small droplet sizes may be associated with health issues. - These systems usually provide less chemical stability than structures with a solid lipid or multilayer emulsions. - Limited control over the release of encapsulated compounds compared with more complex structures.
Multiple emulsions	- 2-step procedure: emulsification of primary emulsion + addition of 3rd phase via homogenization (high-shear mixers, microfluidization, ultrasounds). - Low-energy methods (phase inversion).	Micrometric	- Higher ability to protect and control the release of the encapsulated compounds. - Possibility to reduce the overall fat content. - Possibility to encapsulate lipophilic and hydrophilic within the same system, especially interesting for compounds with synergic effect.	- Highly susceptible to breakdown during storage or when exposed to environmental stresses such as mechanical forces, thermal processing, etc. - More difficult and expensive to prepare than conventional emulsions. - The osmotic balance among the internal and external phases needs to be accurately controlled to avoid compound transfer.
Multilayer emulsions	- Obtention of the primary emulsion by homogenization + biopolymer deposition by using layer-by-layer approach.	Nanometric to micrometric	- Improved physical stability to environmental stresses via control of the composition and properties of the interfacial layer. - High chemical stability of encapsulated components. - Greater control over the release rate of encapsulated compounds due to the ability to manipulate the thickness and properties of the interfacial layer. - Ability to trigger release of functional agents in response to specific changes in environmental conditions.	- The formation of stable multilayer emulsions requires careful control over the system composition and preparation procedures in order to avoid droplet aggregation. - More ingredients and processing steps are required compared to conventional emulsion formation. - These systems are usually more diluted than other systems because of the tendency towards flocculation.
Pickering emulsions	- High-shear homogenization. - High-pressure homogenization. - Ultrasounds. - Microfluidization. - Membrane emulsification.	Nanometric to micrometric	- Droplets are stabilised by solid particles, avoiding the use of surfactants. - High control over the release of encapsulated compound, their contents can be controlled under the action of external factors such as ultrasonic waves. - Shells covering the emulsion droplets in these systems have been found to be even when they undergo high stress	- Modification performed on the solid particle stabiliser to adjust particle wettability can be expensive, time-consuming, and partially effective. - Some particle stabilisers need to be pre-treated via complex or toxic surface modification to be able to function as hydrophobic colloidal stabiliser.
Solid-lipid nanoparticles (SLN)	- High-energy (high-pressure homogenization, sonication, microfluidization). - Low-energy methods (self-emulsification, phase inversion, membrane emulsification).	50 nm–1 µm	- Improved chemical stability of labile compounds by trapping them within a structured solid matrix. - High ability to control the delivery of lipophilic functional components. - Possibility to obtain stable emulsion-based systems containing crystalline lipophilic components. - These promote oral bioavailability of the entrapped compounds through selective lymphatic uptake.	- SLN must be prepared at elevated temperatures to avoid crystallisation of the lipid phase during the homogenization process, which can promote the degradation of heat-sensitive lipophilic components. - The highly ordered crystalline structure can cause compound expulsion. - The lipid used should present a high degree of saturation which can have implications to human health.
Nanostructured lipid carriers (NLC)	- High-energy (high-pressure homogenization, sonication, microfluidization). - Low-energy methods (self-emulsification, phase inversion, membrane emulsification).	10 nm–0.5 µm	- The partial crystal structure facilitates more space to accommodate bioactive ingredients, leading to less expulsion and higher loading capacity and controlled release of encapsulated carotenoid compounds. - Higher encapsulation efficiency and high stability. - High chemical stability of the encapsulated compounds.	- Most formulations include synthetic lipids or surfactants which can be associated with health issues. - There is a lack of a proper method and scaleup techniques.

Adapted from [65,86,87].

## 4. Natural-Based Stabilisers for Emulsion-Based Delivery Systems

Emulsifiers play two key roles in the obtention of emulsion-based systems: emulsion formation and emulsion stabilisation. For emulsion formation, they need to be rapidly adsorbed, decrease the interfacial tensions, and facilitate droplet breakup. Moreover, emulsion stabilisers have to generate strong repulsive forces and provide a resistant interfacial layer, preventing droplet aggregation [88]. A hydrophilic–lipophilic balance (HLB) value is assigned to each emulsifier, which indicates its solubility in oil and/or water phases. Usually, a surfactant with a low HLB value (3–6) is predominantly hydrophobic and is more likely to stabilise W/O emulsions. In contrast, a surfactant with a high HLB number (10–18) is predominantly hydrophilic and better stabilises O/W emulsions. Finally, surfactants with an intermediate HLB number (7–9) have no particular preference for either oil or water [89].

In recent years, synthetic emulsifiers have been the most used to produce emulsion-based delivery systems for bioactive compounds, as these molecules can be rapidly adsorbed at the interface, efficiently reduce the interfacial tension, and provide systems with high stability [90,91,92]. Nevertheless, the consumption of these synthetic emulsifiers might induce health problems and may cause toxic symptoms after long administration periods [93]. For that reason, researchers focus on emulsion stabiliser ingredients from natural sources, which can be classified depending on their chemical structure in proteins, phospholipids, polysaccharides, or saponins.

### 4.1. Proteins

Most proteins from natural sources present an amphiphilic structure since they contain a mixture of polar and non-polar amino acids, which means that can be adsorbed into oil–water interfaces stabilising lipid droplets in emulsions. These emulsifiers tend to be bulkier and diffuse slower to the interface than small molecule emulsifiers, and higher concentrations are needed rather than with smaller molecular weight. However, once at the interface, they provide a strong viscoelastic film that resists mechanical stresses and provides electrostatic and steric stabilisation [94]. Nevertheless, these natural emulsifiers have been found to be highly affected by pH changes and high ionic strength, which can cause bridging flocculation of droplets [95,96]. Regarding natural proteins, whey proteins and caseins from bovine milk have been widely used as emulsifiers, as they are effective for the stabilisation of emulsion-based systems [97,98,99]. Recently, some researchers have focused on the use of plant-based proteins such as those from peas, lentils, or rice, to stabilise emulsion-based delivery systems since they are better for human health, the environment, and animal welfare [100]. As an example, some authors have reported that despite being a poorly soluble protein, pea protein can be used to stabilise vitamin-D-loaded nanoemulsions after a pH-shifting and sonication treatment [101]. In this work, the authors reported small particle sizes < 150 nm and high UV radiation stability of vitamin D3. This highlights that the functionality of these molecules as emulsifiers can be improved by treating them before incorporating them into the delivery systems. Alternatively, rice bran protein was used as an emulsifier of quercetin-loaded nanoemulsions achieving reduced particle sizes (200 nm) and showing relatively high stability [102]. In addition, recent studies have investigated the emulsifying capacity of proteins from algae such as *Nannochloropsis gaditana*, *Tetraselmis impellucida*, and *Arthrospira platensis* [103,104,105]. In these works, proteins extracted from algae were shown to form stable emulsions at similar concentrations to proteins from other sources such as dairy or legumes. Indeed, the minimum particle size that was achieved was observed to be similar when comparing algae proteins to those from milk [103]. Moreover, emulsions containing a protein-rich extract from *Arthrospira platensis* as an emulsifier were shown to present a good emulsifying capacity and provided emulsions with physical stability for up to 30 days. Thus, the use of protein-rich algae extracts as emulsifiers presents an added value since the proteins that they contain can act as emulsion stabilisers, but they also contain great amounts of bioactive compounds.

### 4.2. Phospholipids

Phospholipids have non-polar and polar regions within the same molecule, so they are amphiphilic molecules that can adsorb to oil–water interfaces and stabilise lipid droplets. Phospholipid-based emulsifiers used in the food industry are usually called lecithins. This emulsifier type, which is a major component of cell membranes, can be obtained from both vegetal and animal sources. However, most of the research focused on emulsion-based delivery systems has been performed by using lecithins from vegetal sources, mainly soybean, sunflower, and cottonseed. The HLB of lecithins can be different depending on the phospholipid composition, but the values are usually approximately 8. This means that these emulsifiers can stabilise both O/W and W/O interfaces. Moreover, lecithins stabilise emulsion-based systems via electrostatic repulsion, so when they are adsorbed at the interface, they provide highly negative charges. As an example, Gao et al. [106] observed extremely negative ζ-potential (−70 mV) and particle sizes < 250 nm when soy lecithin was used at concentrations higher than 2% in nanoemulsions that were based on fractionated coconut oil. Moreover, this emulsifier type has been found to be highly effective in reducing the interfacial tension. Indeed, soy lecithin has been found to be more effective than whey protein or gum Arabic in reducing the interfacial tension, showing the lowest particle size when preparing oil-in-water nanoemulsions that encapsulate paprika oleoresin (<140 nm) [97]. Moreover, these authors reported that lecithin nanoemulsions were highly stable when exposed to temperatures (40–80 °C) but were affected by the ionic strength, showing an increase in the particle size and loss of negative electrical charge. Lecithin nanoemulsions have been shown to be stable at a wide range of pH values, presenting no instability phenomena for 7 days at various studied pH values [107]. Indeed, some authors have reported that lecithin emulsions presented a low particle size (<200 nm) at a pH range of 3–8 and a negative ζ-potential, especially at a pH > 4, which was about −60 mV [96]. Moreover, by using this emulsifier over 1% w/w, long-term stable nanoemulsions (up to 86 days) were obtained, which were able to efficiently entrap curcumin within, preventing its autoxidation and, hence, maintaining the antioxidant capacity of the bioactive compound [108]. Soybean lecithin has been found to be also effective in stabilising the oil–water interface of double emulsions. Indeed, by using this emulsifier, emulsions with a particle size of about 4 µm and a phycocyanin encapsulation efficiency of 82% were achieved [109]. Therefore, lecithins seem to be a highly valuable emulsifier since they are highly efficient in reducing interfacial tension and providing systems with high stability over time. Moreover, emulsion-based delivery systems containing these emulsifiers seem to be more stable to external factors such as pH or temperature compared to others such as proteins. 

### 4.3. Polysaccharides 

Some polysaccharides from natural sources can also be useful as emulsifiers since they present an amphiphilic structure that can adsorb at the water-in-oil interface and help to stabilise the system [110]. Moreover, most of them are of vegetal origin, so they can be used in plant-based products. This type of emulsifiers generally present good pH, salt concentration, and temperature stability, but they need to be used in higher amounts to stabilise emulsion-based systems and produce small particles due to their large molecular weight and dimensions [89]. When polysaccharides are adsorbed at the interface, they form relatively thick layers that provide steric repulsion, so they are less affected by changes in pH and ionic strength than proteins [88]. Among them, Arabic gum has been widely used and has been shown to reduce interfacial tension, providing emulsions with particle sizes < 1 µm. However, this polysaccharide seems to be less effective in reducing the particle size and preventing the degradation of the encapsulated carotenoids than others such as whey protein or lecithin [97]. Nevertheless, it provides emulsions with better flocculation stability at different pH values, high ionic strength, and high temperatures than those containing whey protein as an emulsifier due to their steric stabilising mechanism [111]. Therefore, it seems that polysaccharides such as Arabic gum can be potential emulsifiers to obtain stable systems against external factors but present some disadvantages, such as the low stability of the encapsulated compound and higher particle sizes when compared with proteins or phospholipids. Moreover, a natural hydrocolloid exudated by the bark of *Cercidium praecox* tree (Brea gum) has been found to produce emulsions with even more stability than Arabic gum at the same concentration, which was attributed mainly to its higher viscosity [112]. 

Another polysaccharide that is widely used in the food industry is pectin, which has been reported to present emulsifying properties, although the particle sizes that were achieved were not in the range of nanoemulsion [113]. However, a recent work has reported that extracts from avocado residues (from peel and seeds) that are rich in phenolic compounds presented a higher interfacial activity than that of low-methoxyl pectin [114]. Thus, this work demonstrated the advantages of agrifood residues as a source of polysaccharides with emulsifying properties but with added value due to the high content of bioactive compounds that reduced lipid oxidation. In the same way, polysaccharides isolated from seaweed have also been tested as emulsifiers that are rich in bioactive compounds. As an example, polysaccharides from alga *Ulva fasciata* have been tested as emulsifiers in β-carotene-loaded emulsions, showing particle sizes of about 0.8 µm and <10% of encapsulated compound degradation for 4 days at 4 °C [115]. Other algae polysaccharides such as fucoidan have been found to have a good emulsifying capacity, especially when isolated by using microwaves, presenting also antioxidant activity [116,117]. This polysaccharide has shown to form emulsions with higher stability and fucoxanthin encapsulation efficiency than Arabic gum [118]. Moreover, it has been used in combination with other biopolymer, forming complexes. As an example, Jamshidi et al. [119] used whey protein–inulin–fucoidan complexes to stabilise double emulsions and concluded that the presence of fucoidan had a significant influence on the nutritional quality and oxidative stability.

### 4.4. Saponins

Saponins are relatively small amphiphilic molecules that are mostly obtained from plants and that consist of a hydrophobic aglycone and a hydrophilic sugar moiety [120]. These plant-based emulsifiers appear to be highly effective at forming small droplets that are stable over a wide range of conditions (pH, ionic strength, and temperature) [96]. These emulsifiers, which have been shown to provide steric and electrostatic stabilisation, can form interfacial layers with a high dilatational elasticity, inhibiting droplet deformation and coalescence. Among them, saponins obtained from the bark of the *Quillaja saponaria* tree have been shown to reduce the interfacial tension in the oil–water interface faster and to a higher extent than other emulsifiers such as lecithin, whey protein, or Arabic gum, rendering to emulsions with a smaller particle size [121]. The use of this emulsifier has been compared with saponins extracted from other plants: *Tribulus terrestris, Trigonella foenum-graecum,* and *Ruscus aculeatus* [122]. These authors reported the best results by using the *Tribulus terrestris* extract and highlighted the use of saponin-rich extracts as potential emulsifiers due to their similar or even additional functional properties than saponin pure forms, avoiding complex extraction and purification treatments. In another work, by using tea saponin extract from *Camellia lutchuensis* (51.8 wt% saponin content) stable emulsions were obtained in a pH range of 3–9 and thermal processing from 30 °C to 90 °C [123]. 

## 5. Functionality of Emulsion-Based Delivery Systems Containing Natural Emulsifiers

### 5.1. In Vitro Lipid Digestibility and Bioactive Compound Bioaccessibility 

The lipid digestibility of emulsion-based delivery systems has a strong impact on the release and bioaccessibility of the encapsulated compounds. In that sense, it has been found that the emulsifier type can modulate the digestibility of emulsions. Depending on the emulsifier used, emulsions may present different initial properties (particle size, ζ-potential, viscosity, etc.), and their stability may vary during their pass through the gastrointestinal tract, affecting lipid digestibility [124]. 

In recent years, the study of lipid digestibility and compound bioaccessibility in emulsions formulated by using natural emulsifiers has increased. Some authors have compared the use of natural emulsifiers with that of synthetics in the stability of emulsions and their gastrointestinal behaviour (Table 2). As an example, Lamothe et al. [125] compared the digestibility of emulsions containing synthetic emulsifiers (cetyltrimethylammonium bromide (CTAB), Citrem) with that of natural emulsifiers (sodium caseinate, fish gelatin, Arabic gum, modified starch). These authors concluded that, by using some natural emulsifiers such as Arabic gum or sodium caseinate, the digestibility of emulsions was higher than by using synthetic emulsifiers such as CTAB or Citrem. In another work, soybean lecithin was shown to be more effective than synthetic emulsifiers such as Tween 20 or sucrose palmitate in increasing β-carotene bioaccessibility due to its contribution to the formation of mixed micelles and its solubilisation capacity [126]. In contrast, Tan et al. [127] observed that lipid digestibility was higher when synthetic Tween 20 or quillaja saponin were used, compared to the other studied natural emulsifiers (lysolecithin, caseinate, or Arabic gum). However, these authors reported that emulsions containing caseinate presented the same β-carotene bioaccessibility as those with Tween 20 (about 60%), which was attributed to the ability of that protein to inhibit oxidation. These results highlight that bioaccessibility depends not only on lipid digestibility but also on other aspects such as the protection of the compound against degradation or its incorporation into the mixed micelles. The use of gypenosides as a natural emulsifier has been compared to that of Tween 20 in emulsions encapsulating astaxanthin. In that work, gypenoside-emulsion presented lower digestibility and astaxanthin bioaccessibility than emulsion with Tween 20, although the stability of the compound was the same for both emulsions [128]. The authors attributed the lower bioaccessibility when using the natural emulsifier to the lower digestibility and the possible inhibition of micelle formation due to the presence of gypenoside molecules. In double emulsions, novel natural emulsifiers have also been tested as a substitute for those synthetics. As an example, black bean protein (BBP) has been proposed as an effective emulsifier to increase the bioaccessibility of insulin and quercetin by 2.6- and 4.56-fold, respectively, compared to unencapsulated compounds [66]. Moreover, these authors found that BBP-emulsion showed the same lipid digestibility as the emulsion formulated with synthetic Tween 80.

Many authors have based their research on the use of natural emulsifiers, especially on those of plant origin (Table 2). For instance, the use of plant-based emulsifiers such as Arabic gum and quillaja saponin has been compared with that of one animal-based emulsifier (whey protein isolate) in emulsions enclosing vitamin E [129]. The results showed that lipid digestion was equal for emulsions containing whey protein or Arabic gum but lower for those with saponins. The authors correlated the results to the high surface activity of saponins, which may have inhibited their removal by bile acids and lipase. Moreover, the highest Vitamin E bioaccessibility was achieved by using the animal emulsifier (85%), followed by plant-based emulsifiers (65%). In another work, curcumin-loaded nanoemulsions containing soybean lecithin or whey protein as emulsifiers presented the same lipid digestibility. However, the bioaccessibility of the encapsulated compound was higher when whey protein was used than when soybean lecithin was used, which was attributed to the capacity of the proteins to better prevent the degradation of curcumin during digestion [130]. Comparison among different plant-based emulsifiers has also been performed by Yan et al. [131], who studied the digestibility of emulsions formulated with soybean lecithin (SBL) or hydrolysed rice glutelin (HRG) as emulsifiers. These authors reported that SBL-emulsion was more stable against flocculation under gastric conditions and presented higher digestibility than HRG-emulsion under intestinal conditions. In the same way, other authors have studied the lipid digestion of emulsions containing Arabic gum, ghatti gum, or sugar beet pectin as emulsifiers [132]. In this work, most Arabic gum-stabilised droplets were digested, but the extensive flocculation and coalescence of undigested droplets were observed in emulsions stabilised with the two other studied emulsifiers. Moreover, the authors attributed the differences in digestion rate among emulsions to their stability in the stimulated intestinal juice and the resistance of the interfacial layer against displacement by bile salts. In addition, emulsions that contained polysaccharides from alga *Ulva fasciata* presented higher digestibility and β-carotene bioaccessibility than emulsions that were formulated by using other polysaccharides such as Arabic gum or beet pectin, which was attributed to the small particle sizes of the former in the intestinal environment [115]. 

Thus, these studies reveal that the use of emulsifiers of natural origin allows obtaining emulsion-based delivery systems with a functionality comparable to that of systems formulated with synthetic emulsifiers. In addition, differences are observed among the natural emulsifiers studied, as they present diverse properties depending on their structure and origin. Nevertheless, it seems that those of plant origin are not as effective as those from milk in increasing the bioaccessibility of encapsulated compounds, although minimal research has been performed with plant-derived emulsifiers.

**Table 2:** Recent studies on the lipid digestibility and compound bioaccessibility of emulsion-based delivery systems formulated with natural emulsifiers.

System Type	Emulsifiers Used	Encapsulated Compound	Main Findings	Reference
O/W emulsion	Soybean lecithin (SBL), hydrolysed rice glutelin (HRG)	None	SBL-emulsion was more stable against flocculation under gastric conditions and presented higher digestibility than HRG-emulsion.	[131]
O/W emulsion	Lysolecithin (LL), Arabic gum (AG), caseinate (SC), quillaja saponin (QS), Tween 20 (T20)	β-carotene	Digestibility was lower for the emulsions stabilised by LL or SC, than those stabilised by AG, QS, or T20. β-carotene bioaccessibility increased in the following order: LL < AG < SC < QS < T20.	[127]
O/W emulsion	Quillaja saponin (QS), Arabic gum (AG), whey protein isolate (WPI)	Vitamin E	Lipid digestion was slower in QS-emulsions, presumably because the high surface activity of saponins inhibited their removal by bile acids and lipase. Vitamin E bioaccessibility was higher in WPI- than in QS- or AG-emulsions.	[129]
O/W emulsion	Cetyltrimethylammonium bromide (CTAB), Citrem, sodium caseinate (SC), fish gelatin (FG), Arabic gum (AG), or modified starch (MS)	Linseed oil (rich in omega-3 PUFA)	Emulsions prepared with CTAB and GA were the most stable under gastric conditions, while those stabilised by proteins (SC or FG) and MS showed aggregation with partial coalescence in the gastric phase. AG-emulsion showed the highest FFA extent, followed by CTAB- and SC- emulsions.	[125]
O/W emulsion	Arabic gum (AG), ghatti gum (GG), or sugar beet pectin (SBP)	None	The digestion rate decreased in the following order: AG > SBP > GG. Differences were attributed to the stability of the emulsified lipid droplets in the stimulated intestinal juice and the resistance of interfacial layer against displacement by bile salts.	[132]
O/W emulsion	*Ulva fasciata* polysaccharide (UFP), Arabic gum (AG), or beet pectin (BP)	β-carotene	UFP-stabilised emulsion showed higher release extent of free fatty acids and bioaccessibility of carotenoids compared to BP and AG-stabilised emulsions.	[115]
O/W emulsion	Tween 80 (TW), phosphatidylcholine (PC), or citrus pectin (CP)	β-carotene	T80-emulsion presented a higher β-carotene bioaccessibility than those with PC or CP, and it was associated with the higher concentration of incorporated MAG and FFA into the micellar fraction by using T80-emulsion.	[133]
W/O/W double emulsion	Lecithin (L), pectin (P), black bean protein (BBP), or Tween 80 (T80)	Insulin and quercetin	The BBP-stabilised double emulsion presented the lowest particle size during the GIT digestion. Moreover, it yielded a 2.60- and 4.56-fold increase in the bioaccessibility of insulin and quercetin, respectively, by increasing their chemical stability and solubility under simulated gastrointestinal conditions.	[66]
W/O/W double emulsion	gelatin-epigallocatechin gallate (EGCG)-high methoxyl pectin ternary complex	Vitamin C	Gelatin-EGCG-high methoxyl pectin ternary complex had a better protective effect on vitamin C in the internal aqueous phase during in vitro simulated digestion. Compared with the W_1_/O primary emulsion, the double emulsion effectively improved the bioavailability of vitamin C.	[67]
Multilayer emulsion	Quillaja saponin (QS), chitosan (CS), pectin (P)	Astaxanthin	Coating layers of CS and P improved the lipid stability during gastrointestinal digestion and reduced the release of free fatty acids (by nearly 20%). Meanwhile, the release of Astaxanthin was prolonged in the small intestine, and its final bioaccessibility was improved by the coating layers.	[134]
Multilayer emulsion	Sodium caseinate (SC), sulphated fucan (SF), ι-carrageenan (ICA), κ-carrageenan (KCA), or alginate (ALG)	None	All studied multilayer emulsions presented an increased digestibility compared to the primary emulsion. Moreover, the digestion rate and degree of multilayer emulsions decreased in the order of KCA > ALG ≈ ICA > SF.	[135]
Pickering emulsion	Nanochitin (NCh)	Vitamin D_3_	NCh–Pickering emulsions presented lower digestibility and vitamin bioaccessibility than T80-emulsions as a consequence of flocculation, hindered access for lipase to reach lipid, and precipitation of mixed micelles.	[136]
Pickering emulsion	Chitosan (CS)	Roasted coffee oil	CS nanoparticles were shown to be able to adsorb onto oil droplet surfaces, providing efficiency in encapsulating and protecting bioactive compounds during lipid digestion and increasing the bioaccessibility of phenolic compounds.	[137]
Pickering emulsion	Nanofibrillated cellulose (NFC) or whey protein isolate (WPI)	Astaxanthin	Pickering emulsions containing 0.7% NFC presented higher compound stability during digestion than emulsions with 0.7% WPI. However, they presented the same astaxanthin bioaccessibility due to the reduced digestibility of NFC emulsions.	[138]

### 5.2. In Vivo Bioavailability

In vitro studies are very helpful for obtaining information about the expected gastrointestinal stability and digestibility of emulsion-based delivery systems, as well as the bioaccessibility of the encapsulated compound. However, in vivo studies are essential to obtain stronger conclusions and information about the absorption and metabolism of the encapsulated compound. For all that, some authors have investigated the oral bioavailability of different compounds enclosed in emulsion-based delivery systems. To date, most research that has been conducted to increase the bioavailability of bioactive compounds such as curcumin [139], β-carotene [140], or resveratrol [141] by using different emulsion-based delivery systems has been performed by using synthetic emulsifiers. However, in the last years, in vivo research has been focused on emulsions formulated by using emulsifiers of natural origin (Table 3).

Among the emulsifiers of natural origin, those from milk have been widely used to study the in vivo bioavailability of bioactive compounds enclosed in different systems (Table 3). As an example, the use of different β-carotene-loaded emulsion delivery systems containing whey protein as an emulsifier has been investigated [142]. The authors found that the bioavailability of β-carotene was higher when using nanoemulsions than when using macroemulsions or non-encapsulated β-carotene due to their greater capacity to promote the transportation and absorption of the compound in the digestive tract. In the same way, the carotenoid bioavailability was also increased by using excipient emulsions containing sodium caseinate as an emulsifier [11]. Moreover, this study revealed that by increasing the oil content from 0.2 to 1 g, the carotenoid concentration in the plasma of rats increased from ≈ 40 ng/mL to ≈ 110 ng/mL. Therefore, it indicates that emulsifiers from milk are effective in increasing the bioavailability of lipophilic compounds such as carotenoids and that the oil content and the particle size have an effect on their bioavailability. Moreover, curcumin oral bioavailability was increased by five-fold compared to the suspension, when adding chitosan and carboxymethyl konjac glucomannan to a whey-protein-stabilised emulsion to form a multilayer emulsion. In contrast, when the primary emulsion was used, curcumin oral bioavailability was only 1.95-fold greater than when the suspension was used [143]. However, interest in plant-derived emulsifiers has increased in response to demands from consumers with milk protein allergies or intolerances or those with a vegan diet.

Plant-based emulsifiers such as soybean lecithin have been incorporated into nanoemulsions designed to increase the bioavailability of carotenoids from microalga *Dunaliella Salina* (Table 3) [59]. In this study, nanoemulsions containing soybean lecithin increased the bioavailability of carotenoids compared to the control suspension, but better results were observed when whey proteins (by 2.8-fold) rather than soybean lecithin (by 2.15-fold) were used. Other vegetal emulsifiers such as pea protein have been used in combination with chitosan in the formulation of EPA-loaded Pickering emulsions, and these vegetal emulsifiers were shown to be more effective in increasing the bioavailability of the enclosed compound than an emulsion containing Tween 80 [144]. The use of saponins has been investigated in emulsions and nanoemulsions enclosing cholecalciferol (vitamin D3) or α-tocopherol (Vitamin E) that were orally administered to rodents. Parthasarathi et al. [145] found that, by reducing the particle size of emulsions, the bioavailability of α-tocopherol was enhanced, being three-fold higher in rats fed with the saponin-nanoemulsion rather than in those fed with the conventional saponin-emulsion. Moreover, saponin emulsions and nanoemulsions have been shown to increase the bioavailability of vitamin D3 by 36% and 73%, respectively [61], highlighting the relevance of emulsion particle size in increasing the bioavailability of closed compounds.

**Table 3:** Recent studies on the in vivo bioavailability of bioactive compounds enclosed in different emulsion-based delivery systems containing natural emulsifiers.

Bioactive Compound	Dose	System Type	Animal Model	Ingredients	Outcomes	Reference
β-carotene	1 mg/kg BW	O/W nanoemulsion	mice	10% corn oil; 2% whey protein isolate	Nanoemulsions increased transportation and absorption of β-carotene in the digestive tract compared to macroemulsions.	[142]
	60 mg/kg BW	O/W nanoemulsion	rat	30% corn oil; 12% whey protein isolate; or soybean lecithin	Nanoemulsions containing protein-based emulsifiers better increased the bioavailability of β-carotene than those containing soybean lecithin.	[59]
Carotenoids (from fresh spinach puree)	1.0, 0.6, 0.2, and 0 g/kg BW	O/W nanoemulsion (excipient)	rat	10% oil (medium-chain triglyceride and long-chain triglyceride 1:1); 1% sodium caseinate	Carotenoid bioavailability was enhanced by increasing the lipid content due to the higher transfer efficiency of the carotenoids from spinach to fat droplets and mixed micelles.	[11]
Cholecalciferol (VD_3_)	4000 IU kg^−1^ supplementation	O/W emulsion or nanoemulsion	mice	10% corn oil; 2% quillaja saponin	Nano-based delivery system improved the bioavailability and homogeneity of VD absorption.	[61]
Coenzyme Q10	30 mg/kg BW	O/W nanoemulsion	rat	10% soybean oil; 1–10% lecithin	Incorporation of Coenzyme Q10 to nanoemulsions increased the bioavailability of the bioactive compound by 1.8-fold.	[146]
Tangeretin	100 mg/kg BW	O/W emulsion	rat	20% medium-chain triglyceride oil, whey protein concentrate, and gum Arabic, or cinnamaldehyde, or hydroxypropyl methylcellulose	Tangeretin bioavailability increased from 4- to 20-fold after encapsulation, especially in the presence of hydroxypropyl methylcellulose.	[147]
α-tocopherol	100 mg/kg BW	O/W emulsion, submicron emulsion and nanoemulsion	rat	10% sunflower oil; 0.1% saponins	By reducing the particle size of emulsions, the bioavailability of α-tocopherol was enhanced, which was 3 times higher when the nanoemulsion was used than when the emulsion was used.	[145]
EPA	60 mg/kg BW	Pickering emulsion	mice	60% oil; 4% pea protein–chitosan nanoparticles	EPA-loaded Pickering emulsions containing pea protein —chitosan nanoparticles were shown to be more effective in increasing EPA bioavailability than an emulsion containing Tween 80.	[144]
Curcumin	12 mg/kg BW	Multilayer emulsion	mice	10% medium chain oil and 90% WPI aqueous solution (1%), 0.2% chitosan (CS), and 0.1 carboxymethyl konjac glucomannan (CKG)	Emulsions coater with CKG or CS + CKG conferred a higher C_max_ value and improved the bioavailability of curcumin by up to 5-fold compared with free curcumin.	[143]

BW: body weight.

## 6. Concluding Remarks and Future Perspectives 

In recent years, much research has been performed to obtain bioactive compounds from alternative sources that contribute to food sustainability. Among them, microalgae and agrifood residues appear to be promising sustainable sources of pure or rich extracts to be used as ingredients to design novel functional foods. Moreover, novel green extraction methods are being developed to obtain these valuable compounds in a more safe and environmental-friendly way. Various encapsulation systems are being designed to protect these valuable compounds, with emulsion-based delivery systems being effective systems to increase their stability, bioaccessibility, and oral bioavailability. In general, the selection of the emulsion-based delivery system depends on the properties of the compound and the required functionality. Among the existing methods, novel systems such as Pickering emulsions or NLC have been found to provide higher chemical stability and higher control over the release of the encapsulated compounds compared to other systems such as conventional emulsions.

Some of the most recent in vitro and in vivo studies have yielded results that reveal that emulsifiers from natural sources can be used as substitutes for those synthetics, improving the bioaccessibility and oral bioavailability of encapsulated compounds. In addition, although vegetal emulsifiers seem to provide emulsions with characteristics comparable to those of milk proteins, in vitro studies reveal that milk proteins seem to be more effective at increasing the bioaccessibility and bioavailability of the encapsulated bioactive compounds than those of vegetable origin. Nevertheless, most in vivo studies have been performed by using emulsions or nanoemulsions. Therefore, more research is required to evaluate the functionality of those more novel and complex systems containing ingredients from natural sources. Furthermore, in recent years, promising emulsifier molecules of plant and algae origin have been identified but have not yet been studied in vivo. Therefore, more studies are needed to determine the potential of these plant-derived molecules to increase the oral bioavailability of compounds with relevant biological properties.

## Data Availability

The data used to support the findings of this study can be provided by the corresponding author upon request.
